# Ectopic Papillary Thyroid Cancer with Distant Metastasis

**DOI:** 10.1155/2018/8956712

**Published:** 2018-07-11

**Authors:** Oscar R. Vázquez, Frieda Silva, Eduardo Acosta-Pumarejo, Maria L. Marín

**Affiliations:** ^1^Nuclear Medicine Section, Radiological Sciences Department, University of Puerto Rico, San Juan, Puerto Rico, USA; ^2^Radiology Department, VA Caribbean Healthcare System, San Juan, Puerto Rico, USA; ^3^Pathology Department, Isaac Gonzalez Oncologic Hospital, San Juan, Puerto Rico, USA

## Abstract

Ectopic thyroid tissue is a rare clinical entity wherein malignant lesions may arise, the most common one being papillary carcinoma. We present a case of a 68-year-old female who presented with a growing mass in the right clavicle. An MR of the shoulder showed a soft tissue mass arising from the anterior margin of the right distal clavicle. A fine needle aspiration of the mass showed papillary thyroid carcinoma. PET/CT confirmed the clavicular and mediastinal mass. Excision of the clavicular mass and total thyroidectomy with modified right neck dissection were performed. Pathology revealed no evidence of malignancy in the thyroid; lymph nodes were positive for metastatic thyroid carcinoma. Postsurgery CT showed the superior mediastinal mass with surrounding adenopathy; radioiodine (RAI) treatment with dose of 142.1 mCi [5257.7 MBq] was recommended. Posttherapy whole-body scan (WBS) showed RAI avid tissue in the neck and superior mediastinum. Follow-up chest CT revealed pulmonary nodules that increased in number and size; a second RAI therapeutic dose was given. The posttherapy RAI WBS was negative. Repeat PET/CT showed multiple hypermetabolic lesions in the mediastinum, neck, lymph nodes, pulmonary nodes, and right shoulder. The FDG avid lesions with no RAI uptake suggested tumor dedifferentiation.

## 1. Introduction

Ectopic thyroid tissue is a rare clinical entity, with a prevalence of 1: 300,000 in the general population. The ectopic tissue may be located anywhere from the base of the tongue to the diaphragm, the most frequent sites being lingual, thyroglossal, laryngotracheal, and lateral cervical regions. It may also occur in less frequent sites such as the esophagus, mediastinum, heart, adrenal glands, and pancreas [1]. The mediastinum is the most frequent location after the neck [2].

Ectopic thyroid tissue can undergo the same pathologic processes of a normal thyroid gland. Even when the probability of cancer on ectopic tissue is less than 1%, malignant lesions may arise. Differentiated carcinoma accounts for the majority of these tumors, papillary carcinoma being the most common. As in the native thyroid, papillary tumors are the more frequent malignant lesions in ectopic tissue. Lymph node metastasis is common; distant metastases can occur in 10 % of cases. Most of papillary tumors are radioiodine avid and have an excellent prognosis [3]. Lymph node metastases in malignant ectopic lesions are present in 30% of cases.

We present a case of ectopic thyroid tissue in the mediastinum with papillary carcinoma that presented with an unusual aggressive behavior.

## 2. Case Summary

**Figure 1:**
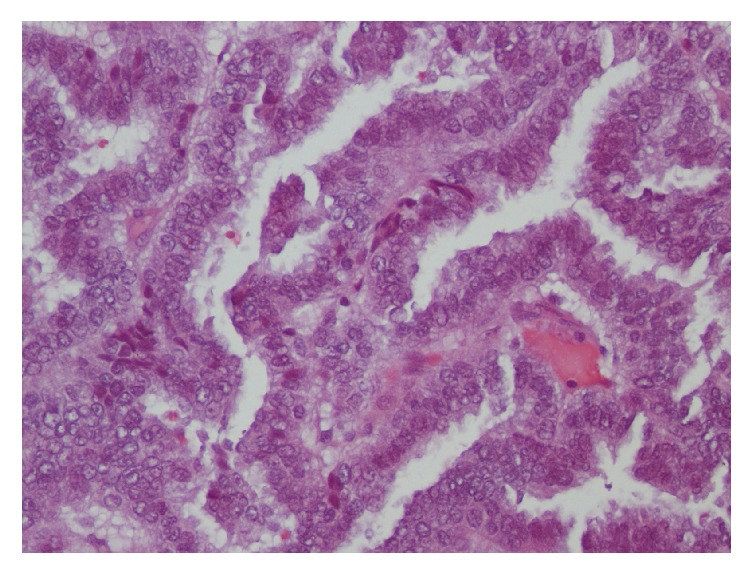
Surgical pathology of the right clavicular mass revealing metastatic papillary adenocarcinoma.

**Figure 2:**
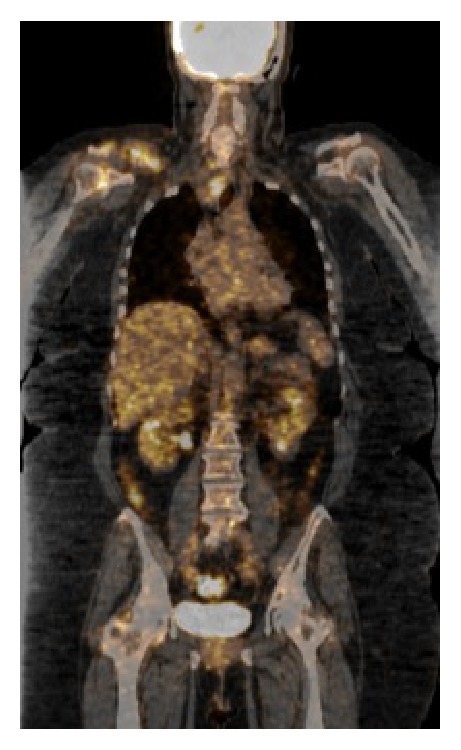
Coronal-fused 18F-FDG PET/CT shows FDG avid lesion in the distal right clavicle with osseous destruction and an FDG avid mediastinal mass displacing the trachea to the left.

A 68-year-old female patient with history of hypothyroidism came to the clinic for evaluation because of a growing mass in the right distal clavicular lesion. An MR of the shoulder region was performed revealing a 5.5 cm soft tissue mass arising from the anterior margin of the right distal clavicle. A fine needle aspiration of the mass was performed; histology showed papillary thyroid carcinoma [Figure 1]. Immunohistochemistry was positive for TTF-1, thyroglobulin, CK19, CK7, and EMA. A whole-body 18F-FDG PET-CT study was ordered. The study was done with 15.5 mCi [576 MBq] of 18F-FDG and it showed a hypermetabolic lesion in the distal right clavicle with evidence of osseous destruction and enlarged retro- and infraclavicular lymph nodes [SUV max: 12.5 g/mL]. There was also an FDG avid mediastinal mass displacing the trachea and esophagus [SUV max: 7.9 g/mL]. The thyroid gland was small and atrophic [Figure 2].

**Figure 3:**
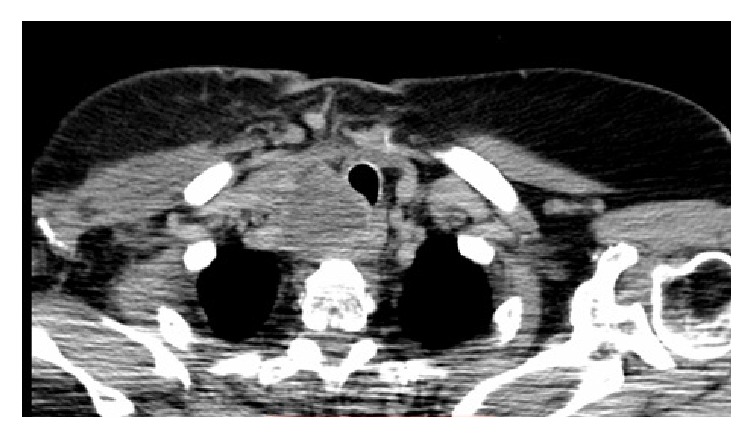
Axial chest CT showing a hypodense, solid, infrahyoid mass extending to the superior mediastinum.

Complete excision of the right clavicular mass and the distal clavicle was performed. This was followed by a total thyroidectomy, with modified right neck dissection of levels 3, 4, and 5. The pathology report of the thyroid gland was negative for malignancy; however, the neck dissection procedure was positive for metastatic papillary thyroid carcinoma in level III cervical lymph nodes. Postsurgery CT scan showed a hypodense, solid, infrahyoid mass extending to the superior mediastinum with surrounding adenopathy [Figure 3].

**Figure 4:**
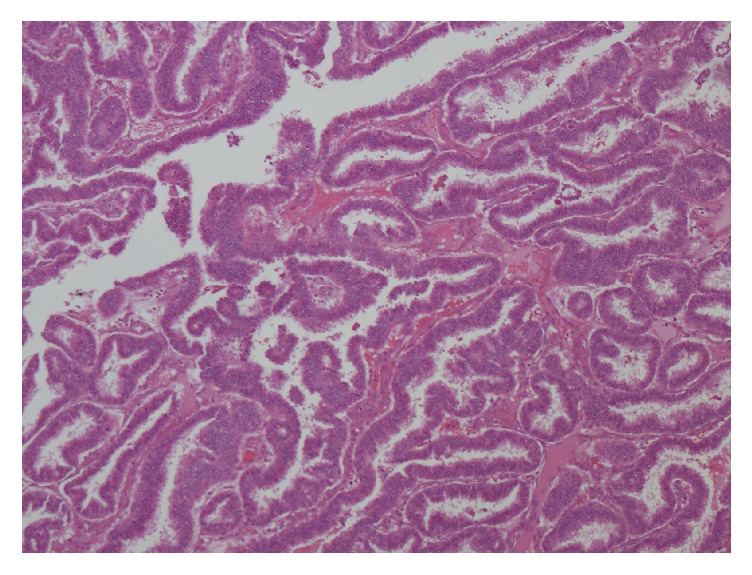
Pathology report of central neck biopsy of superior mediastinal mass revealing papillary thyroid carcinoma.

**Figure 5:**
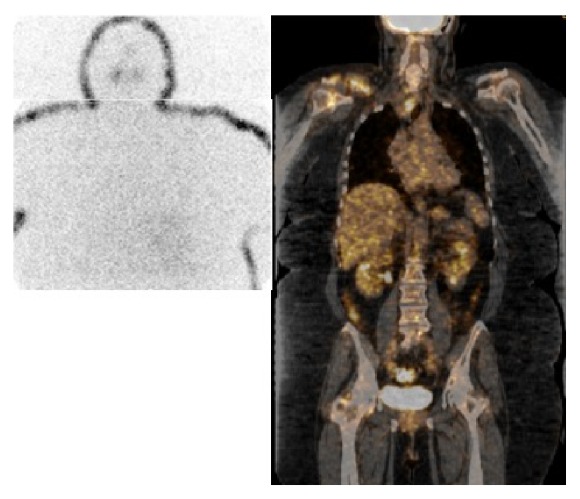
Left: posttherapy 131-iodine WBS shows no evidence of functional thyroid tissue. Right: coronal-fused images of 18F-FDG PET/CT study performed after posttherapy WBS show the FDG avid mediastinal and right shoulder masses.

The patient was subsequently treated with external beam radiation therapy to the distal clavicular region. Central neck biopsy of the superior mediastinal mass was performed. The biopsy reported papillary carcinoma, with similar cellular characteristics as that reported in the clavicular mass; surgical pathology report revealed papillary thyroid carcinoma with tumor cells positive for TTF-1 and focally positive for thyroglobulin [Figure 4]. Due to the morbidity of the procedure, surgical excision of the mediastinal mass was not recommended at the moment. Instead, the patient was referred to the nuclear medicine service for evaluation with a 131-iodine whole-body scan (WBS) and possible treatment with radioiodine (RAI). The study was performed with 3 mCi [111 MBq] of 131-iodine [TSH level: 42.6 uU/mL; stimulated Tg: 66.4 ng/mL; TgAb: 3.8]. It revealed abnormal tracer uptake in the region of the thyroid bed and upper mediastinum. The patient was then prepared for RAI adjuvant therapy for residual thyroid tissue. A dose of 142.1 mCi [5257.7 MBq] of 131-iodine was administered and the posttherapy WBS revealed focal tracer uptake in the lower anterior neck extending to the thoracic inlet and superior mediastinum, consistent with persistent functional thyroid tissue; no focal RAI uptake was observed in the region of the right clavicle. Neck sonogram and CT scan of the neck and superior mediastinum with IV contrast performed afterwards showed multiple abnormal lymph nodes in the thyroid bed and neck arguing in favor of recurrent or metastatic disease. The CT also showed additional pulmonary nodules and interval size decrease of the superior mediastinal mass. The patient was reevaluated and a second dose of RAI was recommended to be given three [3] months after the contrast enhanced CT. A dose of 172.1 mCi [6367.7 MBq] of 131-iodine was administered [TSH level 74.3 uIU/mL]; at this time, the stimulated Tg was 1,216.6 ng/mL and the TgAb < 1 IU/mL. The posttherapy WBS showed no evidence of radioiodine concentration in the thyroid bed, mediastinum, supraclavicular area, or neck. Follow-up PET/CT performed with 15 mCi [555 MBq] of 18F-FDG showed the previously described mediastinal FDG avid mass, with a cystic or necrotic area, development of hypermetabolic neck lymph nodes, axillary nodes, and pulmonary nodes and a large mass in the right shoulder area [SUV max: 5.5] [Figure 5]. At this time the tumor was considered to be dedifferentiated and patient was started on tyrosine kinase inhibitor therapy with good short term therapeutic response.

## 3. Discussion

The thyroid gland develops around the 4^th^ week of embryonal development and originates from a diverticulum located in the median ventral wall of the pharynx, between the first and second pharyngeal pouches dorsal to the aortic sac [4]. The primitive thyroid tissue penetrates the underlying mesenchymal tissue and descends anterior to the hyoid bone and laryngeal cartilages until it reaches the lower neck. This process takes three weeks and is usually complete by the seventh week of gestation. Abnormal descent of the thyroid tissue may occur and gives rise to ectopic thyroid tissue, which can be found anywhere from the base of the tongue to the diaphragm. The probability of cancer on ectopic thyroid tissue is less than 1% and when it occurs papillary carcinoma is the most common histology [5–7].

Several criteria have been described to establish the presence of ectopic thyroid tissue in the mediastinum versus a substernal or a retrosternal extension of the thyroid gland. These are as follows: the tissue has blood supply from intrathoracic vessels rather than from cervical arteries, there is a normal or absent [without history of surgery] cervical thyroid gland, and the cervical thyroid gland does not have a similar pathologic process as the ectopic tissue and has no history or evidence of documented malignancy [5, 8].

This case presented meets the above-mentioned criteria of an ectopic tissue. The native thyroid was negative for malignancy and the thyroid gland had no intrathoracic extension as demonstrated in the CT. Unfortunately, the mediastinal mass was not excised, and we could not demonstrate the intrathoracic source of the blood supply. However, the patient had no bleeding complications during the excision of the thyroid gland. Furthermore, the tumor had an aggressive evolution, initially presenting with a clavicular mass and bone involvement. This makes the case a very unusual one, since as stated before most malignant ectopic thyroid tissue presents a papillary histology, usually not aggressive in nature.

The whole-body scan performed after the first radioiodine therapy failed to reveal radioiodine uptake in the clavicular region, the mediastinal masses, or the lung lesions. Tumors with high thyroglobulin levels, negative radioiodine scan, and FDG avidity tend to be more aggressive and dedifferentiated. PET studies have been used for several years in such cases for diagnostic and prognostic purposes. PET/CT may change patient management in up to 40% of cases. The volume of the FDG avid lesions and the SUV were considered strong predictors of survival. Overall survival and cancer specific survival have been reported to be 8.9 and 9.6 years, respectively, in radioiodine refractory thyroid cancer. The time of tumor dedifferentiation was found to be the second most important prognostic factor for the survival. Patients with less than 3 years of tumor dedifferentiation had a worse prognosis [3, 9, 10].

In our case, the elapsed time for dedifferentiation was 6 months. There was no uptake in the mediastinal and clavicular masses in the posttherapy radioiodine study, suggesting the tumor had dedifferentiated and furthermore became more aggressive in nature. The whole-body scan performed after the second therapeutic dose of radioiodine did not reveal any foci of abnormal RAI uptake, further confirming the loss of the sodium iodine symporter to concentrate iodine, as a sign of dedifferentiation.

To our knowledge, this is the first report of an unusually aggressive ectopic thyroid tumor. There are several important facts in the presentation and evolution of the case that should raise the suspicion of a possible dedifferentiated aggressive ectopic tumor. The presence of early distant metastasis, the rapidly rising thyroglobulin levels, and the poor radioiodine uptake are ominous signs in thyroid malignancies. The early identification of bad prognostic signs is important in the therapy planning and follow-up.

## 4. Conclusion

Ectopic thyroid tissue is a rare entity. The chances of harboring malignant cells are low. When malignant lesions occur, they are usually papillary carcinomas. Even when the incidence of thyroid carcinoma has been increasing in the last years, the mortality has not changed, except in radioiodine refractory tumors.

Tumoral lesions in ectopic mediastinal lesions are a rare entity; an aggressive papillary carcinoma is even less common. To our knowledge, this is the first report of a widely metastatic tumor in ectopic thyroid tissue. It is important to keep in mind that tumor dedifferentiation may occur in ectopic tumors in order to provide patients with early therapeutic options.
